# Fat digestibility is reduced in old cats with subnormal cobalamin concentrations

**DOI:** 10.1017/jns.2014.59

**Published:** 2014-12-30

**Authors:** Anna Salas, Carmen-Loreto Manuelian, Marta Garganté, Núria Sanchez, Sonia Fernández, Marco Compagnucci, Jose Joaquín Cerón, Isabelle Jeusette, Lluís Vilaseca, Celina Torre

**Affiliations:** 1 R&D Department, Affinity Petcare, 08174, Sant Cugat del Vallès, Barcelona, Spain; 2 Doc's Veterinary Clinic, 08338 Premià, Barcelona, Spain; 3 Animal Medicine and Surgery Department, Veterinary School, Campus of Excelence Mare Nostrum, University of Murcia, 30100 Espinardo, Murcia, Spain

**Keywords:** Feline nutrition, Cobalamin, Ageing, Digestibility, fPL, feline pancreatic lipase, IBD, inflammatory bowel disease, MA, middle-aged

## Abstract

Fat digestibility is decreased in old cats for unknown reasons. Subclinical gastrointestinal diseases and pancreatic dysfunction, both related to ageing, can affect food digestibility. The aim of the present study was to elucidate the prevalence of subnormal cobalamin concentration and pancreatic disease in old cats and study the relationship between both markers and fat digestibility. A total of sixty-four cats without evident signs of gastrointestinal disease were included and grouped according to age: (1) fifteen middle-aged (MA), aged 3–7 years; and (2) forty-nine old, aged 10–17 years. All cats were tested for serum cobalamin, specific feline pancreatic lipase (fPL) and feline trypsin-like immunoreactivity. Then, sixteen of the old cats were selected and grouped according to cobalamin and fPL concentrations: control (normal cobalamin and fPL); low vitamin B_12_ (cobalamin <290 ng/l; normal fPL); and high fPL (normal cobalamin; fPL >4 µg/ml). A food digestibility trial with a high-fat diet (21·6 %) was performed. In the old group, cobalamin was lower and fPL higher than in MA cats. Of the old cats (*n* 49), 14 % had subnormal cobalamin, 8 % had a severe increase in fPL, 2 % had both alterations and 14 % had a slight increase in fPL. By contrast, MA cats did not have cobalamin deficiency or an increase in fPL concentrations. Fat digestibility was lower in low vitamin B_12_ cats than control cats. Decreased fat digestibility is not present in all old cats but could be a characteristic of subclinical chronic gastrointestinal disease. Cobalamin concentration, as a marker of gastrointestinal disease, could be useful for the routine evaluation of old cats.

Nutrient digestibility and absorption decrease in humans and rats with age^(^^1^^,^^2^^)^. Similarly, as occurs in humans, both protein and fat digestibilities are decreased in old cats, but fat seems to be the most affected nutrient digestibility^(^^3^^,^^4^^)^. In a study by Anantharaman-Barr *et al.*^(^^3^^)^, digestibility comparison between young (1 year), middle-aged (MA) (2–5 years) and old (>10 years) cats showed a reduction of 9 % in fat digestibility of old cats compared with MA cats. Peachey *et al.*^(^^5^^)^ reported greater apparent total fat digestibility in young cats (3 years old; 94·4 %) than in old cats (11 years old; 92·2 %). This reduction in fat digestibility with ageing has been described as not necessarily being related to clinical conditions, as it has been described also in healthy cats, but it is still not clear how ageing affects fat digestibility^(^^6^^)^. Impairment of gastrointestinal and pancreatic function with age is a possible cause to be explored. This would be consistent with observations that most chronic gastrointestinal diseases of cats are more frequent in MA and old cats. For example, chronic pancreatitis occurrence has been shown to be significantly correlated with age^(^^7^^)^. The prevalence of chronic pancreatitis in apparently healthy cats was reported to be 45 % through postmortem histopathological examinations, suggesting that mild pancreatic inflammation might not be clinically evident^(^^7^^)^.

Cobalamin is a water-soluble vitamin with an important function in many biochemical reactions, including DNA synthesis, methionine synthesis from homocysteine and conversion of propionyl into succinyl coenzyme A from methylmalonate. Deficiency in cobalamin leads to notable metabolic and clinical disorders. In cats, absorption of cobalamin is particularly complex and mainly dependent on the function of the pancreas and small intestine. As a consequence, the main causes for hypocobalaminaemia have been postulated to be exocrine pancreatic insufficiency, chronic pancreatitis, extrahepatic biliary disease, intestinal bacterial competition and ileal mucosal disease^(^^8^^)^. Low concentrations of serum cobalamin have been described in cats with gastrointestinal disease^(^^9^^)^, and data have shown that cobalamin-deficient cats are significantly older than control cats^(^^10^^)^.

Old cats are prone to have mild chronic pancreatitis and other gastrointestinal diseases, and although these diseases may not be clinically evident, they may have a relationship with cobalamin serum concentrations and this could also be related to decreased digestibility of macronutrients as a consequence of ageing.

To elucidate the relationship between these factors, we analysed a MA and old population of cats for specific feline pancreatic lipase (fPL) as a marker of pancreatitis and cobalaminaemia. Nutrient digestibility was tested in old cats with elevated concentrations of fPL and reduced concentrations of serum cobalamin.

## Materials and methods

The Ethics Committee for Animal Wellbeing at Affinity Petcare approved all experimental procedures.

### Animals and blood analyses

A total of sixty-four mixed-breed, neutered cats from a private colony were included in the study and grouped according to age: fifteen MA cats (aged 3–7 years) and forty-nine old cats (aged 10–17 years). The health of all cats was assessed by a veterinarian before inclusion, and no signs of gastrointestinal disease (vomiting, diarrhoea, anorexia or abdominal pain) were reported. Cats were fed with different commercial complete dry diets, all of them supplemented with cyanocobalamin (100–170 µg/kg DM; 5–9 fold National Research Council requirements). Body weight was determined and blood samples were taken after a 12 h fasting period. Serum biochemistry and complete blood count analyses were carried out at IDEXX Laboratories (Barcelona, Spain). Serum fPL was analysed with a commercial ELISA IDEXX kit (Spec fPL^TM^ Test Kit). Feline trypsin-like immunoreactivity was determined in the Gastrointestinal Laboratory at Texas A&M University using a RIA technique (normal range is considered when lower than 3·5 µg/l, slight increase between 3·6–5·3 µg/l, and severe increase for values higher than 5·4 µg/l). Both cobalamin and folate concentrations were determined at the Murcia Veterinary School using a solid-phase, competitive chemiluminescent enzyme immunoassay, according to the manufacturer's instructions (Immulite 2000; Siemens Healthcare Diagnostics). The laboratory reported normal ranges of cobalamin for values <290 ng/l and of folate between 9·7 and 21·6 ng/ml. Abdominal ultrasonography (General Electric Logiq E) was performed on cats with subnormal cobalamin concentration to evaluate signs of pancreatic and gastrointestinal inflammation. Ultrasound guided fine needle aspiration cytology was performed whenever needed for the diagnosis. Samples were analysed at Histovet Laboratories (St Quirze del Vallès, Barcelona, Spain).

Of the old cats, sixteen (aged 11·8 (sem 0·6) years) were assigned to three groups for food digestibility tests according to their fPL and cobalamin (vitamin B_12_) serum concentrations: control (vitamin B_12_ and fPL concentrations in the reference range; *n* 5; range 10–12 years); low vitamin B_12_ (vitamin B_12_ < 290 ng/l and fPL in the reference range; *n* 5; range 10–14 years); and high fPL (vitamin B_12_ in the reference range and fPL > 4 µg/l; *n* 6; range 10–15 years).

### Digestibility test protocol

The digestibility assay was carried out through a quantitative collection of faeces, according to AAFCO guidelines (www.aafco.org). All cats from the three groups included in the digestibility test received the same commercial dry high-fat diet with 10 % fat coming from lard (% DM: 42·3 % protein, 21·6 % fat, 1·2 % crude fibre, 27 % N-free extracts; Advance Cat Kitten, Affinity Petcare).

During a diet adaptation phase of 7 d the cats were housed together in pens, and during the following faecal collection phase over 7 d the cats were individually housed in stainless-steel cages which were maintained at a constant temperature (21°C) and light-controlled (14 h light–10 h dark) throughout the study period. Food and water were available *ad libitum* throughout the duration of the study. Body weight was recorded at day 1, day 8 and day 14 and food intake was recorded daily when the cats were individually housed.

### Laboratory analyses of food and faeces

During the last 5 d of the collection phase, faeces were collected daily and dried for 24 h in a 90°C air oven. Afterwards, faeces from each cat were pooled, ground and homogenised. Food samples were also ground and homogenised. Both diet and faeces were analysed following official methods for ash (AOAC 942.05^(^^11^^)^), crude protein (Dumas, AOAC 990.03^(^^11^^)^), fat (acid hydrolysis, AOAC 954.02^(^^11^^)^) and moisture (air oven 103°/4 h, EC No. 152/2009)^(^^12^^)^ analysis.

### Digestibility calculations

Apparent total gastrointestinal tract digestibility of the nutrients was calculated following the formula: ((nutrient intake on DM basis (g/d) – faecal output on DM basis (g/d))/(nutrient intake on DM basis (g/d))) × 100^(^^13^^)^. Digestible energy was calculated from digestible nutrients (digestible energy = 39·29 kJ/g of digestible fat + 23·83 kJ/g of digestible protein + 17·14 kJ/g of digestible N-free extract) and metabolisable energy was estimated by subtracting 3·76 kJ per g of digestible crude protein as proposed in 2006 by the National Research Council^(^^14^^)^.

### Statistical analyses

Data from analyses (normally distributed) were statistically analysed using Student's *t* test to compare between old and MA cats and different states of cobalaminaemia, and the Pearson correlation was used to study correlations between age and markers. Significance was established at *P* < 0·05.

To analyse digestibility results between groups, data were statistically analysed with Kruskal–Wallis one-way ANOVA and *post hoc* analyses were performed with the Mann–Whitney *U* test incorporating Bonferroni correction to perform multiple comparisons between groups since data were not normally distributed. Significance was established at *P* < 0·05.

The PASW Statistics 17.0 statistical software package was used for all analyses (SPSS Inc.)

## Results

Cats in the old group had significantly higher blood urea (MA: 16·4 (sem 0·7) *v*. old: 21·8 (sem 1·1) mmol/l; *P* = 0·008) and P concentrations (MA: 36 (sem 1) *v.* old: 43 (sem 1) mg/l; *P* = 0·008) and a lower concentration of serum albumin (MA: 31 (sem 1) *v.* old: 28 (sem 0·4) g/l; *P* = 0·014) than MA cats. All these values were in the normal range reported by the laboratory.

Concentration of serum cobalamin in old cats was lower (MA: 1108 (sem 67) ng/l; old: 755 (sem 53) ng/l; *P* = 0·001) and fPL was higher (MA: 2·2 (sem 0·15) µg/l; old: 2·9 (sem 0·18) µg/l; *P* = 0·004) compared with MA cats.

Of the old cats, 14 % (seven of forty-nine; four male and three female) had subnormal cobalamin concentrations (<290 ng/l), 8 % (four of forty-nine; two males and two female) had a severe increase in fPL consistent with pancreatitis (>5·4 µg/l), 2 % (one of forty-nine; female) had both alterations, and 14 % (seven of forty-nine; three male and four female) had a slight increase in fPL (3·6–5·3 µg/l). By contrast, MA cats did not have subnormal cobalamin concentrations or an increase in serum fPL. None of the cats had trypsin-like immunoreactivity consistent with exocrine pancreatic insufficiency (<12 µg/l). Serum folate concentrations in the cats were all within the reference range.

Considering all cats (*n* 64), cobalamin concentration negatively correlated with age (*r* –0·54; *P* < 0·001) and fPL (*r* –0·3; *P* < 0·05). Age was significantly higher and body weight significantly lower in cats with subnormal cobalaminaemia (<290 ng/l; age: 12·8 (sem 0·7) years; body weight: 3947 (sem 441) g) compared with cats with cobalamin within the reference range (>290 ng/l; age: 9·6 (sem 0·4) years; body weight: 5051 (sem 181) g).

The digestibility tests performed with old cats showed that DM food intake and faecal output (both on a DM basis and as-is basis) were not significantly different between groups (Table 1). Cats with low vitamin B_12_ concentrations had decreased fat digestibility (*P* = 0·009) and lower measured metabolisable energy of the diet (*P* = 0·009) than the control cats. Crude protein and N-free extract digestibilities were not different between the cats with low vitamin B_12_ concentrations and control cats. No significant differences were observed in nutrient digestibilities between control and high fPL groups or between groups with low vitamin B_12_ and high fPL, even though the control group showed the highest digestibility values for all the nutrients and metabolisable energy (Table 1). Mean body weight and daily food intake were not significantly different between the groups during the test, and none of the groups significantly changed their body weight during the digestibility test (Table 1).

**Table 1.** Digestibility test with old cats fed a high-fat diet: cats with normal cobalamin and feline pancreatic lipase (fPL) (controls), cats with subnormal cobalamin concentrations (low vitamin B_12_) and cats with high fPL (Mean values with their standard errors; *n* 16)

	Control (*n* 5)		Low vitamin B_12_ (*n* 5)		High fPL (*n* 6)		
	Mean	sem	Mean	sem	Mean	sem	*P*†
Initial BW (g)	5314	534	3476	210	4963	632	0·103
Final BW (g)	5384	587	3498	231	5085	568	0·078
Food intake, DM basis (g/d)	47·1	5·7	46·6	2·1	46·6	7·1	0·836
Food intake, DM basis (g/kg mBW)	13·2	1·0	18·5*	1·1	14·4	2·2	0·065
Faecal output, as-is basis (g/d)	16·4	2·6	26·8	4·5	18·9	2·4	0·127
Faecal output, DM basis (g/d)	6·2	0·86	8·3	0·79	7·3	0·99	0·327
Apparent total digestibility (%)							
DM	87·7	0·37	83·0	1·00	83·9	2·0	0·153
OM	91·2	0·27	86·9	0·98	88·1	1·4	0·067
CP	89·8	0·37	86·3	1·4	86·8	1·9	0·311
Fat (acid hydrolysis)	92·9	0·71	83·9*	3·1	89·0	2·1	0·024
NFE	94·7	0·31	93·2	0·74	92·2	1·2	0·279
Metabolisable energy (kJ/kg DM)	19 961	58	18 825*	267	19 258	293	0·029

BW, body weight; mBW, metabolic body weight; OM, organic matter; CP, crude protein; NFE, N-free extracts.

* Mean value was significantly different from that of the control group (*P* < 0·05; Mann–Whitney *U* test with Bonferroni correction).

† Significance of the Kruskall–Wallis one-way ANOVA.

Ultrasonography was conducted in six of the cats with subnormal cobalamin concentrations. Cytology was performed in those cats with echographic changes compatible with chronic pancreatitis to rule out other diagnoses. In all cases, cytology results were compatible with an inflammatory process. In these cats where muscular layer hypertrophy was seen using ultrasonography, we could not distinguish between intestinal lymphoma and inflammatory bowel disease (IBD) since biopsies were not allowed by the ethics committee in cats. Gastritis was considered when wall thickening was observed using ultrasonography. Cholangitis was considered when the image showed dilated biliary ducts. One cat with both abnormal cobalamin and fPL showed ecographic changes compatible with chronic pancreatitis (confirmed by cytology), IBD/lymphoma and cholangitis. The other cats with subnormal cobalamin concentrations showed ecographic images and cytology compatible with IBD/lymphoma (*n* 1), with IBD/lymphoma and chronic pancreatitis (*n* 2), with IBD/lymphoma and gastritis (*n* 1) and with IBD/lymphoma and cholangitis (*n* 1).

## Discussion

Ageing is not considered as a disease but as a life stage with several gradual changes in metabolic activity, reduced homeostasis capacity of the organism and an increase in age-related conditions^(^^15^^)^. In the present study, when comparing MA and old cats, we reported mild changes in renal markers (blood urea and P) but no differences were observed in creatinine concentrations and there were no signs of chronic kidney disease (creatinine <141·4 µmol/l; <1·6 mg/dl). Moreover, despite both groups of cats having albumin concentrations in the reference range (24–40 g/l), the lower concentrations of albumin observed in old compared with MA cats could be attributed to preclinical kidney dysfunction or a compromised intestinal absorption, since liver enzymes (aspartate transaminase (AST) and alanine transaminase (ALT)) were not different between the groups.

There is some contradictory data on the prevalence of hypocobalaminaemia in cats. While Ibarrola *et al.*^(^^16^^)^ found that cobalamin deficiency is uncommon in the UK, Reed *et al.*^(^^9^^)^ reported 16·5 % of cases of hypocobalaminaemia in cats with gastrointestinal disease. Data from cats in the USA showed a significantly higher presence of hypocobalaminaemia with confirmed gastrointestinal disease (61 %)^(^^10^^)^. A recent study in the UK reported a prevalence of 28 % in cats with gastrointestinal signs but a lack of correlation between serum cobalamin concentrations and histological scores or clinical signs^(^^8^^)^. In the present study, the prevalence of subnormal cobalaminaemia in cats with no evident signs of gastrointestinal disease was about 14 %, and so should not be considered a rare occurrence.

Cobalamin deficiency has been mainly attributed to pancreatic disease (exocrine pancreatic insufficiency or pancreatitis) or intestinal disease^(^^17^^)^. Of the cats we included in the study, none of them had serum trypsin-like immunoreactivity concentrations indicating exocrine pancreatic insufficiency, and only one cat had both cobalamin and fPL concentrations outside the reference ranges. These results suggested that hypocobalaminaemia in the asymptomatic cats could be attributed to gastrointestinal disease rather than just to pancreatic insufficiency. Bacterial overgrowth could be a cause of hypocobalaminaemia, but this was ruled out in the present study because all cats with subnormal cobalamin concentrations had normal serum folate. Ultrasonography showed that the main cause for this deficiency is an intestinal disease, and in some cats it occurred with alterations in the pancreas and stomach or cholangitis. These are among the diseases reported by Simpson *et al.*^(^^10^^)^ as the main causes of subnormal cobalamin concentrations. The main difference in the present study compared with others is that the cats included had no evident signs of gastrointestinal disease, suggesting that serum concentrations of cobalamin could be a very sensitive marker to be checked in all old cats before the appearance and subsequent treatment of symptoms. The lower body weight observed in cats with subnormal cobalamin concentration could be indicative of a subclinical gastrointestinal disease, although the body condition score of the cats should be checked at the time of enrollment to be able to state whether the cats were in a worse body condition. A limitation in our study could be that the diagnosis of gastrointestinal disease was performed by ultrasonography combined with a cytology examination, when a biopsy would be a better tool to improve the diagnosis. Therefore, other possible causes of the deficiency cannot be ruled out. Specificity of fPL concentration for feline pancreatitis is high (67–100 %)^(^^18^^,^^19^^)^, but sensitivity for mild or chronic feline pancreatitis has been speculated to be lower than for acute pancreatitis^(^^20^^)^; thus false-negative results could occur. In fact, two cats with normal concentrations of fPL showed echographic changes compatible with chronic pancreatitis, and they were confirmed in the cytology.

From digestibility trials we can see how it is mainly the old cats with a low cobalamin status that have difficulties in digesting nutrients, suggesting that perhaps subclinical gastrointestinal disease in these cats can be sufficient to decrease the absorption of some nutrients. Digestibility was affected in cats with subnormal cobalamin concentrations only in terms of fat content of the diet, with a decrease of 9 % fat digestibility compared with the control group. This reduction is similar to that observed in the study of Anantharaman-Barr *et al.*^(^^3^^)^, where fat digestibility was decreased by 9 % in old cats compared with MA cats. Overeating could be causing a reduction in the digestibility of a diet, but the food intake of the cats was not significantly different between the groups, although a statistical trend was shown when food intake was adjusted by metabolic body weight.

Cats with elevated serum fPL showed no differences in any digestibility value; however, we need to clarify that the cats had no clinical signs of disease, but a fPL concentration which could be associated with pancreatitis. However, our intention in the present study was to check if the digestibility capacity of apparently healthy cats could be affected and this is why we included this population. It is likely that different results would be obtained using cats with clinical pancreatitis.

The present study is probably limited by the number of cats in each group in which digestibility was measured. This number needs to be increased for a better understanding of the results. As an example, protein digestibility was not significantly different between the groups, but perhaps with more cats in each group the results would reach significance, since it was found that both low vitamin B_12_ and high fPL groups showed reduced numerical digestibility values compared with control cats. On the other hand, results from the literature are inconsistent in terms of demonstrating a reduction of protein digestibility in old cats; it is likely that maybe the reduction is not as frequent as in fat digestibility.

Old cats (aged ≥10 years) with fPL and cobalamin serum concentrations within the reference range seem to maintain a high digestibility of macronutrients. In contrast, when apparently healthy cats have subnormal cobalamin concentrations, this seems to be related to a reduction in the capacity to digest fat. Conversely, this was not observed in our data when cats had an above-normal concentration of fPL (>4 µg/l) and therefore a possibility of mild pancreatitis.

These results suggest that decreased food digestibility is not present in all old cats as has been proposed previously^(^^21^^)^ but could be a characteristic of cats with subclinical chronic gastrointestinal disease, as indicated by subnormal cobalamin serum concentrations. Moreover, serum cobalamin concentration, as a marker of subclinical gastrointestinal disease, could be used for analysis in the routine evaluation of old cats.
